# “Powerful placebo”: A teaching and learning concept addressing placebo and nocebo effects in competency-based communication training

**DOI:** 10.3205/zma001693

**Published:** 2024-09-16

**Authors:** Hanna Öhlmann, Adriane Icenhour, Sigrid Elsenbruch, Sven Benson

**Affiliations:** 1 Ruhr University Bochum, Department of Medical Psychology and Medical Sociology, Bochum, Germany; 2 University Hospital Essen, University of Duisburg-Essen, Department of Neurology, Center for Translational Neuro- and Behavioral Sciences, Essen, Germany; 3 University Hospital Essen, University of Duisburg-Essen, Institute for Medical Education, Institute of Medical Psychology and Behavioral Immunobiology, Center for Translational Neuro- and Behavioral Sciences, Essen, Germany

**Keywords:** placebo effect, nocebo effect, expectations, communication, physician-patient interaction, competence, NKLM

## Abstract

**Aim:**

Placebo and nocebo effects are based on expectations that are formed by how doctors communicate and can influence the efficacy of medical treatment. Given the implications for doctor-patient communication and the learning objectives listed in NKLM 2.0, we herein present a novel teaching and learning concept to impart competency-based knowledge about placebo and nocebo effects.

**Method:**

The teaching and learning concept was piloted with N=324 third-semester medical students. It combines a self-guided, small-group component to gather communication strategies and apply them in a video-recorded conversation, followed by a classroom-based session to reflect on and discuss the videos and to learn basic scientific and theoretical knowledge. The evaluation involved written feedback from the students and lecturers (structure/process) and an analysis of the videos (students' learning success). To supplement this, the overall course evaluation was included since this new teaching concept was not specifically evaluated by the students.

**Results:**

Course structure and process were rated positively. The active involvement of the students in the subject matter and the balance between theoretical, scientific and practical content was emphasized positively. Analysis of the learning success showed that the students were able to effectively transfer the knowledge gained about placebo and nocebo effects to conversational situations.

**Conclusion:**

The topic of placebo/nocebo is optimally suited to teach communication skills with its many links to knowledge, translational approaches and added value for medical practice. When doing this, video-recorded conversations appear to be an effective tool to achieve learning objectives. This teaching and learning strategy offers possibilities for expanding communication curricula.

## Zusammenfassung

**Zielsetzung:**

Placebo- und Noceboeffekte basieren auf Erwartungen, die durch ärztliche Kommunikation geprägt werden und die Wirksamkeit medizinischer Behandlungen beeinflussen. Angesichts der Implikationen für die ärztliche Gesprächsführung und der im NKLM 2.0 hinterlegten Lernziele wurde ein Lehr-/Lernkonzept entwickelt und evaluiert, das kompetenzorientiert Wissen zu Placebo- und Noceboeffekten vermittelt.

**Methodik:**

Das Lehr-/Lernkonzept wurde bei N=324 Studierende im 3. Fachsemester Medizin erprobt. Es kombiniert eine Selbstlerneinheit in Kleingruppen zur Erarbeitung von Kommunikationsstrategien und deren Anwendung in einer videodokumentierten Gesprächssituation, sowie eine Präsenzeinheit zur Reflektion und Diskussion der Videos und zur Vermittlung wissenschaftlicher Grundlagen. Die Evaluation umfasste schriftliche Rückmeldungen der Lehrenden (Struktur/Prozess) sowie eine Analyse der Videodokumentation (Lernerfolg der Studierenden). Ergänzend wurde die Evaluation des Gesamtkurses herangezogen, da keine gezielte Evaluation des neuen Lehrkonzepts durch Studierende erfolgte.

**Ergebnisse:**

Struktur und Prozess des Kurses wurden seitens der Lehrenden als gut bewertet. Es wurden die aktive Auseinandersetzung der Studierenden mit der Thematik sowie die Balance zwischen theoretischen, wissenschaftlichen und praktischen Inhalten positiv hervorgehoben. Die Lernerfolgsanalyse zeigte, dass die Studierenden erworbenes Wissen zu Placebo- und Noceboeffekten erfolgreich auf Gesprächssituationen übertragen konnten.

**Schlussfolgerung:**

Mit vielfältigen Möglichkeiten zur Wissensverknüpfung, translationalen Ansätzen und Mehrwert für die ärztliche Praxis eignet sich das Thema Placebo/Nocebo optimal für die Vermittlung kommunikativer Kompetenzen. Ein wirksames Instrument zur Erreichung der Lernziele scheinen dabei die videodokumentierten Gesprächssituationen zu bieten. Das Lehr-/Lernkonzept bietet Anregungen zur Erweiterung von Kommunikationscurricula.

## 1. Introduction

Communication skills are a core competency in medical practice [28], [32]. Research on the placebo effect (Latin *placebo* = “I shall please”) very clearly demonstrates how communication can influence not only the expectations of treatment, but also the efficacy and tolerance of drug therapy and medical interventions [7]. Targeted application of strategies that increase expectations makes it possible to positively influence, for example, the effectiveness of post-operative pain therapies following knee replacements and breast cancer surgeries [2], [33], the outcomes of bypass surgeries [31], and the effect of immunosuppressive drugs following organ transplantation [21]. Placebo effects encompass more than just non-specific interferences such as regression to the mean or spontaneous remissions. Rather, they are based on specific effects of expectations in the treatment context. These arise from learning processes and previous experience and are significantly shaped not only by doctor-patient communication, but also in interaction with other participants in the healthcare system [7]. Besides positive expectation effects, the effects of negative expectations can also occur (nocebo effects; Latin *nocebo* = “I will harm”). These can, for instance, be induced by informed consent discussions and influence the occurrence of side effects [14]. Placebo and nocebo effects are based on complex psychoneurobiological mechanisms, which include, among others, the opioidergic, dopaminergic, and cannabinoid systems and changes in activity patterns in the brain and spinal cord [29].

These examples of placebo and nocebo effects outlined above do not only illustrate the importance of communication skills [20] in medical education. They also emphasize direct implications for physician-patient interactions. Placebo and nocebo effects are already present in decision-making processes and thus in conversations regarding prescriptions, yet only few studies exist on how these should be conducted [16]. Placebo and nocebo effects are further relevant to interactions with patients during interventions, for instance, unpleasant or painful procedures (e.g., [34]).

The significance of placebo and nocebo research for medical education and the need for teaching concepts aligned with it have already been identified as part of an expert consensus [5], [10], [12], [23], [27], [30]. The National Catalogue of Competency-based Learning Objectives for Undergraduate Medical Education (NKLM, version 2.0, [https://nklm.de/zend/menu], last accessed on 15 Dec. 2023) contains learning objectives regarding practical and theoretical knowledge, as well as competencies concerning placebo and nocebo effects (VII.3-01.1.9; VIII.2-02.1; VIII.2-02.1.4). In addition to the extensive work done to implement and further develop communication curricula for medical students (see [15], [18], [19]), there is a need for competency-based teaching and learning strategies that impart knowledge about placebo and nocebo effects and underscore the consequences associated with how doctors communicate with patients. However, to the best of our knowledge, not just in Germany but internationally there are neither such strategies for practical implementation nor any reports, including those on integration into existing educational formats. This project report presents the conception, implementation and evaluation of a teaching and learning concept centered around the topic of placebo and nocebo effects.

## 2. Project description

### 2.1. Learning objectives and teaching strategy

Overarching educational goals for teaching communication in Medical Psychology include emphasizing the importance of psychosocial factors to medical practice, theoretical fundamentals and practical skills regarding physician-patient interactions and consultations. Combined with the NKLM 2.0 objectives, these goals provide an important basis for the present project, which was also aimed at giving students theoretical knowledge about placebo and nocebo effects and their underlying mechanisms. In addition to these primarily cognitive, knowledge-based learning objectives, attention was also turned particularly to affective and psycho-motoric learning objectives and the meta-cognitive processes of self-reflection [11], [22]. Focus was placed on raising awareness of the importance of doctor-patient communication in regard to treatment expectations and the resulting clinical results [7], as well as on practicing how positive expectancy effects can be induced and negative ones minimized through communication [3], [14]. To boost student motivation in terms of self-determination theory and through constructive and interactive learning activities according to the ICAP framework of Chi & Wylie [6], phases of self-guided learning and independent work (creating a video-recorded doctor-patient conversation) were designed and then combined with a classroom-based session to teach theoretical knowledge and to discuss and reflect on the videos.

### 2.2. Teaching and learning concept

The German-language course module titled “power of expectation” was developed as part of TRR/SFB289 [https://treatment-expectation.de/en/] and implemented in the third-semester medical psychology and medical sociology curriculum as a required course in the integrated reformed medical degree program at the Ruhr University Bochum. The entire curriculum is comprised of 45 teaching units. A certificate of completion is issued based on regular and active participation in the classroom sessions and the completion of an assignment, meaning no grades are given. The six course modules in the curriculum cover the principles of communication/anamnesis, prevention/patient motivation, conveying bad news, dealing with dementia patients, resilience, and “power of expectation”-the course module presented here. This module was offered for the first time in the 2022/23 winter semester to a cohort of 324 students, who were divided into 34 small groups of 10 students each (groups are fixed during all preclinical semesters and as part of the problem-based learning approach).

### 2.3. Structure and content of the course module

**Figure 1:**
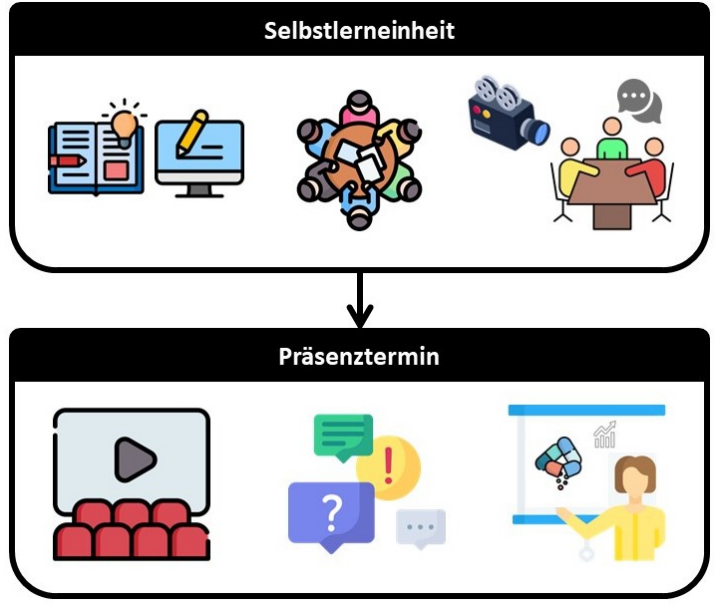
Schematic illustration of the module’s sequence (German version). Self-guided component (“Selbstlerneinheit”): Working in small groups at first, the students individually prepare a written assignment to familiarize themselves with expectancy effects in medicine. After this, the students create a conversational situation involving a physician, patient and a family member that covers all of the relevant aspects, including the initial greeting and saying good-bye, and which is then enacted by the students and video-recorded. During the subsequent classroom-based session (“Präsenztermin”), in which two small groups meet, the two videos serve as a way into a lively discussion about the role of doctor-patient communication in the context of expectancy effects. A lecture on the scientific evidence regarding placebo and nocebo effects and their underlying mechanisms concludes the course module.

The semester-long unit (see figure 1 (Fig. 1)) entailed a self-guided component designed as independent small-group work followed by a classroom-based session. The assignment for the self-guided component was disseminated to the students via the learning platform Moodle at the start of the semester after the introductory session on the Medical Psychology and Medical Sociology curriculum. The assignment consisted of a written and an interactive part. On-time submission of the assignment was the condition under which the course could be successfully completed (preparation time was 10 weeks max., with independent time management). The assignment was not graded, but rather used as teaching material (didactic tool) during the classroom session so that students received direct feedback. The written part was based on two German articles on placebo and nocebo effects arising from physician-patient interaction [1], [14], in which current studies are summarized and recommendations for communication are derived. Based on this, the students were asked to reflect on the effects of different medical statements on patients (e. g., “You are an at-risk patient”, “We will pay special attention to how you respond to the treatment”, “You don't need to be afraid”, “I’ll do everything to make you feel safe“, cited according to [9], [17]). In a written assignment afterward, students explained the approaches and strategies with which placebo effects can be used and encouraged and nocebo effects can be avoided or reduced.

In the interactive part of the self-guided component, students composed a conversation between a doctor, a patient and the patient’s family containing all of the relevant aspects, including the initial greeting and saying good-bye, which could then be turned into a 10- to 12-minute-long video. Based on the conversational context, the assignment gave instructions for a role play that should focus on the personal interactions; it was stated that neither the cinematography nor the medical accuracy of any statements would be graded. The case vignette, meaning the patient's case history and reason for seeking medical care, was the same for all 34 groups (see table 1 (Tab. 1)).

The instruction for half of the groups (17 out of 34 groups) was to depict a conversation that, from a medical perspective, had great potential for tapping into placebo effects and positively influencing the course of treatment (e.g., by creating a safe environment, focusing on positive aspects, building trust through confident demeanor and statements, linking risks with the therapeutic goal, etc.). In contrast, for the other half of the groups, the assignment was to depict a situation that posed a high risk of unleashing nocebo effects and negatively affecting the course of treatment (e.g., emphasizing risks and possible side effects; glossing over information or making false promises, a non-committal communication style covering all eventualities, presenting fears and concerns as unfounded, etc.).

Each final classroom session (approximately 3 hours) was attended by two groups of 10, of which one group had been given the placebo assignment and the other group the nocebo assignment. First, the videos were presented to both groups and discussed in regard to the communication and interactions. Direct comparisons were made and the differences in verbal, paraverbal and nonverbal communication and their effects on the participants were reflected upon. The students were able to experience how the dynamics changed between the doctor and patient. This enabled a discussion about different approaches and statements and their effects on the course of the conversation and the physician’s role regarding a patient's treatment expectations. Building on this, a lecture gave further insights into the scientific evidence on placebo and nocebo effects and the underlying mechanisms (see section 1 above). Together with the students, selected studies were discussed and their results debated, during which ethical aspects, such as the right to self-determination and the medical duty to inform, were also included.

## 3. Results

The first implementation of the concept described here was evaluated in the 2022/23 winter semester in terms of the Kirkpatrick model’s level of reaction (structures/process) and learning success. The results and the underlying methodology are described and discussed in the following.

### 3.1. Learning success: written assignment and video-recorded conversations

The written assignment and the videos were used as indicators of learning success in the self-guided, small-group component. All N=324 students in the third semester of preclinical medical study (see table 2 (Tab. 2) for semester-level age and gender distribution) successfully completed the assignment for the self-guided component in the 34 small groups. There were no student questions about the assignment; all of the assignments were submitted in full and on time. Using the literature that was made available, all of the groups were able to complete the written assignment in detail (average of 4±1 pages) using practical examples that reflected extensive delving into the topic. The video-recorded conversations (average duration 10:41±1:48 min.) showed diverse and creative application of the theoretical content. The students considered not only the conversational style, but also contextual factors and the use of props. For example, anatomic models were used to clearly show and explain medical information. Telephones and laptops were used to simulate distractions and interruptions in the conversation. Listening in on telephone conversations or consultations with other patients was also used to show the influence of indirect information, including the resulting doubt and lack of confidence. Combining other contextual factors like the participants’ seating arrangement, waiting room situations and interactions with the “office staff”, the students were successful in transferring the extensive knowledge about placebo and nocebo effects that they had learned in their groups to the simulated physician-patient interactions.

Furthermore, N=18 videos (9 videos each for the placebo and nocebo assignments) were randomly selected and evaluated according to a structured list of criteria by two trained and blinded raters who did not participate in the project. This list, developed by us, entailed two subscales (placebo, nocebo), based on which the frequency of placebo- or nocebo-associated behaviors (defined according to the literature used in the course; [1], [14]) were recorded. The frequency or occurrence of specific behaviors (e.g., positive/negative suggestions, considering/ignoring previous experiences) was evaluated in each of six categories (formulation of statements, explanation of risks, previous experiences & expectations, attentive care, doctor-patient relationship, closure of the conversation). When evaluating the videos, a high correspondence was seen between the ratings for the placebo and nocebo-inducing behaviors based on the relevant list of criteria and the actual group assignment (placebo/nocebo). In 89% of the cases, both of the raters’ evaluations corresponded with the group assignment. This supports the claim that the students were successful in enacting placebo- or nocebo-inducing behaviors in the video recordings. Good inter-rater reliability was seen for these evaluations for the placebo subscale (ICC=.84, 95% CI [.62, .94], p<.001) and the nocebo subscale (ICC=.87, 95% CI [.68, .95], p<.001).

Which behaviors predominantly contributed to the differentiation between placebo and nocebo videos (and, accordingly, were frequently applied by the students) were further explored. To do this, one-sided Bonferroni-corrected paired t-tests with consideration for the effect size (Cohen’s d) were used to analyze which items were rated significantly higher on the subscale for the group assignment than on the opposite subscale. For the groups given the placebo assignment, compared with the nocebo groups, there were on average significantly higher scores on the placebo subscale for the items previous experiences & expectations (t_(8)_=8.22, p<.001, d=2.74), explanation of risks (t_(8)_= 9.74, p<.001, d=3.25), formulation of statements (t_(8)_=11.03, p<.001, d=3.68), attentive care (t_(8)_=15.5, p<.001, d=5.17), and doctor-patient relationship (t_(8)_=15.64, p<.001, d=5.21). For the groups given the nocebo assignment, compared with the placebo subscale, there were on average significantly higher scores on the nocebo subscale for the items formulation of statements (t_(8)_=3.20, p<.05, d=1.07) and explanation of risks (t_(8)_=4.26, p<.01, d=1.42).

### 3.2. Structure/process (student evaluations of the overall course)

The student evaluations of the entire course in medical psychology & medical sociology, which were done online and voluntarily by students in the integrated reformed medical degree program at the Ruhr University Bochum, served as indicators for structure and process. For this evaluation, the conventional academic grading scale is used to give an overall grade and a grade for the organizational process. In addition, the students state if the course was held on more than 85% of the scheduled dates and if a “narrative thread” was discernible throughout. A total of N=197 students participated in the evaluation. As overall rating, the course in medical psychology & medical sociology received an average grade of 2.41 (“good”). The organizational process was evaluated with an average grade of 1.99. None of the students stated that the course had taken place on fewer than 85% of the scheduled dates. Of the students participating in the evaluation, 9.12% were unable to discern a narrative thread running through the course. Comparisons with the overall course evaluations from the previous year and the following year (2021/22 and 2023/24 winter semesters) did not show any signs of a systematic change in the overall satisfaction of the students; on average, the evaluation for all three years was “good”. This can mean that the introduction of the new module did not significantly affect the overall satisfaction with the course, whereby the possibility must also be considered if, next to any potential cohort effects, there were any minor changes, such as a different lecturer, which could have had an influence. Furthermore, it must be noted as a limitation that nearly without exception the free-text comments contained vaguely formulated comments by the students. Hence, the very few individual comments specifically about the module and/or the module’s lecturers do not allow any further conclusions to be drawn and emphasize the general need for a specific evaluation.

### 3.3. Structure/process (lecturers)

**Figure 2:**
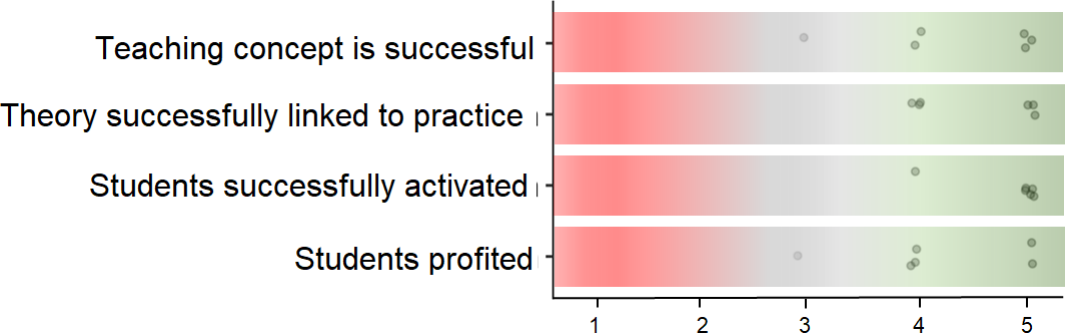
Evaluation of the teaching/learning concept by the instructors (N=6) using closed questions on Likert scale (1=completely disagree, 2=mostly disagree, 3=neutral, 4=mostly agree, 5=completely agree).

The participating lecturers (see table 2 (Tab. 2) for details on sociodemographics) evaluated the course module based on a questionnaire with four closed questions (Likert scale; 1=completely disagree, 5=completely agree). The teaching concept, the goal of activating the students, and the linking of theory to practice were rated predominantly positively. The lecturers also widely agreed that the students were able to benefit from the course unit (see figure 2 (Fig. 2)).

In addition, four open-ended questions were asked about the general impression, positive/negative aspects of implementation, and the suitability of the didactic methods for teaching the course content. Further questions asked about the extent to which the students had been able to link the content on placebo/nocebo with other content concerning doctor-patient communication, whether students had given any feedback, and if there were any additional comments. The free-text comments were analyzed using summative qualitative content analysis [24], [26]. The free-text comments were inductively categorized as part of the qualitative content analysis; frequent assessments and unusual statements were interpreted and cited verbatim (see table 3 (Tab. 3)). In regard to the “students’ individual preparation” during the self-guided component, all six lecturers mentioned that the students took the content of other courses into consideration, five lecturers brought up the good quality of the results, and two also pointed out the productive collaboration among the students. In regard to the “classroom atmosphere”, student interest (N=5 mentions), activation of students (N=3) and the positive atmosphere (N=2) were identified. For the “scientific lecture”, the rather high expectations and the complexity of the studies were criticized (N=4); at the same time, the opportunity to teach scientific material was focused on positively (N=2). In terms of the didactic aspects, the clear “difference between the placebo and nocebo assignments” (N=3) and the successful “balance between theoretical, scientific and practical content” (N=6) were mentioned.

## 4. Discussion

The inclusion of placebo and nocebo effects in medical education is important, not least because of their great relevance to the physician’s conduct and behavior. The significance of expectancy effects in medicine is scientifically well-proven in experimental and clinical studies [7], [8].

Anchoring the topic of placebo/nocebo in NKLM 2.0 supports the position that knowledge as well as competency should be taught in this area. In line with this, the project here pursues primarily affective and psychomotoric, along with cognitive, learning objectives [11], [22] and is meant to encourage self-reflection regarding the importance of doctor-patient communication. Bearing didactic perspectives in mind, a self-guided study phase was placed prior to the classroom session in order to boost motivation, individual responsibility and constructive/interactive learning [6].

Lecturers and students evaluated the project positively overall in terms of structure and process. As a limitation it must be borne in mind that the students’ feedback refers to the entire course on medical psychology & medical sociology and not specifically to the project. In particular, the lecturers rated the activation of the students and the linking of theory to practice positively. The learning success was evaluated on the basis of the video-recorded, doctor-patient conversations. Successful and creative responses by all of the students were seen here, which is meaningful in terms of the intent to activate the students. Successful implementation of the conversational elements related to placebo and nocebo effects and thus an effect on behavior were documented in the evaluations of the conversations by blinded raters.

## 5. Conclusion

In summary, this teaching and learning project gives support to the high potential of treatment expectations as a topic in medical education. Here, in particular, activating practical elements appears to contribute to learning success. This topic offers students the opportunity to apply knowledge and reflect, while connecting translational research approaches and the practice of communicating with patients and, hence, can be a highly relevant addition to existing communication curricula.

## Notes

### Funding

This work was funded by the Deutsche Forschungsgemeinschaft (TRR 289 Treatment Expectation, project number 422744262, sub-project A04 (Elsenbruch) and SC (Benson)).

### Authors’ ORCIDs

- Hanna Öhlmann: [0000-0003-2594-6233]
- Adriane Icenhour: [0000-0001-6323-0960]
- Sigrid Elsenbruch: [0000-0002-6528-2665]
- Sven Benson: [0000-0002-4487-4258]

## Acknowledgements

Thanks to all of the students for their highly diverse work and to all of the participating lecturers for putting this idea into practice and for the evaluation.

## Competing interests

The authors declare that they have no competing interests. 

## Figures and Tables

**Table 1:**
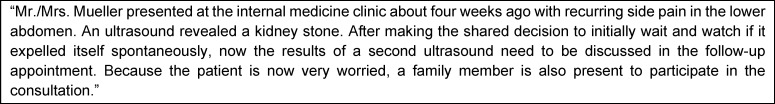
Case vignette

**Table 2:**
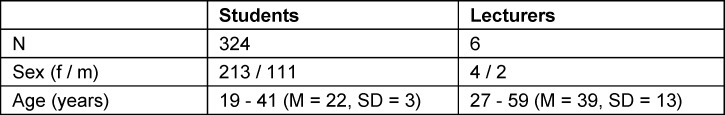
Demographic information

**Table 3:**
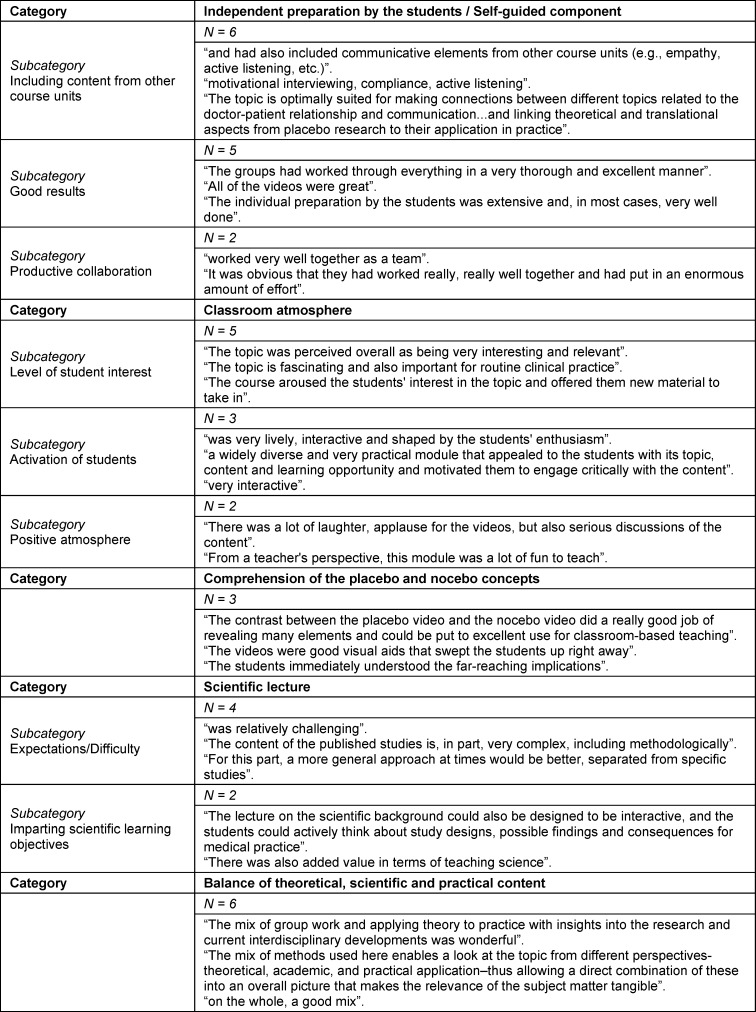
Qualitative content analysis of the evaluation by the lecturers
